# Interim Estimates of 2013–14 Seasonal Influenza Vaccine Effectiveness — United States, February 2014

**Published:** 2014-02-21

**Authors:** Brendan Flannery, Swathi N. Thaker, Jessie Clippard, Arnold S. Monto, Suzanne E. Ohmit, Richard K. Zimmerman, Mary Patricia Nowalk, Manjusha Gaglani, Michael L. Jackson, Lisa A. Jackson, Edward A. Belongia, Huong Q. McLean, LaShondra Berman, Angie Foust, Wendy Sessions, Sarah Spencer, Alicia M. Fry

**Affiliations:** 1 Influenza Division, National Center for Immunization and Respiratory Diseases, CDC; 2 University of Michigan and Henry Ford Health System; 3 University of Pittsburgh Schools of the Health Sciences and UPMC; 4 Baylor Scott and White Health, Texas A&M University Health Science Center College of Medicine; 5 Group Health Research Institute; 6 Marshfield Clinic Research Foundation

In the United States, annual vaccination against seasonal influenza is recommended for all persons aged ≥6 months (1). Each season since 2004–05, CDC has estimated the effectiveness of seasonal influenza vaccine to prevent influenza-associated, medically attended acute respiratory illness (ARI). This report uses data from 2,319 children and adults enrolled in the U.S. Influenza Vaccine Effectiveness (Flu VE) Network during December 2, 2013–January 23, 2014, to estimate an interim adjusted effectiveness of seasonal influenza vaccine for preventing laboratory-confirmed influenza virus infection associated with medically attended ARI. During this period, overall vaccine effectiveness (VE) (adjusted for study site, age, sex, race/ethnicity, self-rated health, and days from illness onset to enrollment) against influenza A and B virus infection associated with medically attended ARI was 61%. The influenza A (H1N1)pdm09 (pH1N1) virus that emerged to cause a pandemic in 2009 accounted for 98% of influenza viruses detected. VE was estimated to be 62% against pH1N1 virus infections and was similar across age groups. As of February 8, 2014, influenza activity remained elevated in the United States, the proportion of persons seeing their health-care provider for influenza-like illness was lower than in early January but remained above the national baseline, and activity still might be increasing in some parts of the country (2). CDC and the Advisory Committee on Immunization Practices routinely recommend that annual influenza vaccination efforts continue as long as influenza viruses are circulating (1). Persons aged ≥6 months who have not yet been vaccinated this season should be vaccinated. Antiviral medications are an important second line of defense to treat influenza illness and should be used as recommended (3) among suspected or confirmed influenza patients, regardless of patient vaccination status. Early antiviral treatment is recommended for persons with suspected influenza with severe or progressive illness (e.g., hospitalized persons) and those at high risk for complications from influenza, no matter how severe the illness.

Methods used by the U.S. Flu VE Network have been published previously (4). At five study sites, patients aged ≥6 months seeking outpatient medical care for an ARI with cough, within 7 days of illness onset, were enrolled.* Study enrollment began after laboratory-confirmed cases of influenza were identified through local surveillance for ≥2 consecutive weeks. Trained study staff members reviewed appointment schedules and lists of symptoms to identify patients with ARI and approached eligible patients (or parents/guardians) to complete a brief screening survey. Patients were eligible for enrollment if they 1) were aged ≥6 months on September 1, 2013, and thus were eligible for vaccination; 2) reported an ARI with cough and onset ≤7 days earlier; and 3) had not been treated with influenza antiviral medication (e.g., oseltamivir) during this illness. Consenting participants completed an enrollment interview. Respiratory specimens were collected from each patient using nasal and oropharyngeal swabs, which were placed together in a single cryovial with viral transport medium. Only nasal swabs were collected for patients aged <2 years. Specimens were tested at U.S. Flu VE Network laboratories using CDC’s real-time reverse transcription polymerase chain reaction (rRT-PCR) protocol for detection and identification of influenza viruses. Participants were considered vaccinated if they received ≥1 dose of any seasonal influenza vaccine ≥14 days before illness onset, according to medical records and registries (at Wisconsin and Washington sites) or medical records and self-report (at Michigan, Pennsylvania, and Texas sites). VE was estimated as 100% × (1 – odds ratio) comparing odds of vaccination among influenza-positive versus influenza-negative participants. Estimates were adjusted for study site, age, sex, race/ethnicity, self-rated health, and days from illness onset to enrollment using logistic regression. Interim VE estimates for the 2013–14 season were based on patients enrolled through January 23, 2014.

Of the 2,319 children and adults with ARI enrolled at the five study sites during December 2, 2013–January 23, 2014, a total of 784 (34%) tested positive for influenza virus by rRT-PCR (Figure); 778 (99%) of these viruses were influenza A, and six (1%) were influenza B (Table 1). Among 755 subtyped influenza A viruses, 742 (98%) were pH1N1 viruses. The proportion of patients with influenza differed by study site, age, race/ethnicity, and interval from onset to enrollment (Table 1). The proportion vaccinated was 38% to 48% across sites and also differed by age, race/ethnicity, and interval from onset to enrollment.

The proportion vaccinated with 2013–14 seasonal influenza vaccine was 29% among influenza cases compared with 50% among influenza-negative controls (Table 2). After adjusting for study site, age, sex, race/ethnicity, self-rated health, and days from illness onset to enrollment, VE against medically attended ARI attributable to influenza was 61% (95% confidence interval [CI] = 52%–68%). The adjusted VE for all ages against medically attended ARI caused by pH1N1 virus infection was 62% (CI = 53%–69%). Similar VE against pH1N1 was observed for all age groups.

## Editorial Note

Interim results for the 2013–14 season indicate that vaccination has reduced the risk for influenza-associated medical visits by approximately 60%, demonstrating the benefits of influenza vaccination during the current season. Influenza activity is likely to continue for several more weeks in the United States. Vaccination efforts should continue as long as influenza viruses are circulating. Persons aged ≥6 months who have not yet received the 2013–14 influenza vaccine should be vaccinated. As of February 8, 2014, approximately 134 million doses of influenza vaccine had been distributed in the United States for the 2013–14 season, from approximately 138–145 million doses that were anticipated to be available for the U.S. market. Because some vaccine providers might have exhausted their vaccine supplies at this time, persons seeking vaccination might need to call more than one provider to locate vaccine.†

These age-adjusted interim VE estimates for the 2013–14 influenza vaccine suggest continued effectiveness in preventing outpatient medical visits associated with pH1N1 virus infection. The 2009 influenza pandemic viruses have continued to circulate each season since the 2009 pandemic, but the 2013–14 influenza season is the first season since 2009–10 during which the pH1N1 viruses have predominated; as of February 8, 2014, pH1N1 viruses accounted for nearly 96% of subtyped influenza A viruses reported to CDC (2). Interim VE estimates for 2013 influenza vaccine for prevention of pH1N1-associated outpatient ARI visits were similar to VE estimates for monovalent pandemic and seasonal influenza vaccines for prevention of outpatient medical visits associated with pH1N1 virus infection during previous influenza seasons (4–7) and are consistent with recent interim estimates from Canada (8). Nationally, more than 99% of pH1N1 viruses tested by CDC this season, including 40 viruses from U.S. Flu VE Network sites, have been antigenically similar to A/California/7/2009, the pH1N1 component of 2013–14 influenza vaccines. In addition, deep sequencing analysis of 43 pH1N1 virus specimens from the Wisconsin site showed genetic similarity to other recent pH1N1 viruses that have been tested and found to be antigenically similar to the recommended vaccine virus (Thomas C. Friedrich, PhD, School of Veterinary Medicine, University of Wisconsin-Madison, unpublished data, 2014).

These interim estimates suggest similar preventive benefits against pH1N1 influenza virus infections across age groups. During the pandemic, young adults, children, pregnant women, and persons with medical conditions (including morbid obesity) that placed them at high risk for influenza-related complications§ experienced high rates of severe illness and influenza-associated hospitalization. Although influenza-associated hospitalization rates during the 2013–14 season have been highest among children aged <5 years and persons aged 50–64 and ≥65 years, as of February 8, 2014, approximately 60% of reported influenza-related hospitalizations have occurred in persons aged 18–64 years, and 22% of reported influenza-related hospitalizations among women of childbearing age (15–44 years) have occurred in pregnant women (2). Interim results indicate significant protection from vaccination among adults aged 18–64 years. However, early estimates for the 2013–14 season¶ indicated that as of mid-November, only 34% of adults aged 18–64 years had received influenza vaccine this season, compared with 41% of children (aged 6 months–17 years) and 62% of adults aged ≥65 years. Among pregnant women, early estimates for the 2013–14 season indicated that only 41% had been vaccinated by mid-November. A study of pregnant women showed that vaccination during the 2010–11 and 2011–12 seasons significantly reduced influenza-associated medical visits (9). Final 2013–14 influenza season vaccination coverage estimates will be available after the end of the season.

As of February 8, 2014, influenza activity remained elevated nationally and widespread across most of the country. These VE estimates imply that some vaccinated persons will become infected with influenza. Clinicians should maintain a high index of suspicion for influenza infection among persons with ARI while influenza activity is ongoing. Early antiviral treatment can reduce influenza-associated illness severity and complications (3). Early antiviral treatment is recommended for persons with suspected influenza with severe or progressive illness (e.g., hospitalized persons) and those at high risk for complications from influenza,** no matter how severe the illness. Antiviral medications should be used as recommended for treatment in patients with suspected influenza, regardless of vaccination status. The decision to initiate antiviral treatment should not wait for laboratory confirmation of influenza and should not be dependent on insensitive assays, such as rapid influenza diagnostic tests.

The findings in this report are subject to at least four limitations. First, vaccination status included self-report at three of five sites; dates of vaccination were available only for persons with documented vaccination obtained from medical records or immunization registries. Verification of vaccination status at all sites will be available for end-of-season VE estimates, which might differ from interim estimates. Second, information from medical records and immunization registries is needed to evaluate VE for fully versus partially vaccinated children (certain children aged <9 years require 2 vaccine doses) and by vaccine type (e.g., inactivated compared with live attenuated), as well as to evaluate the effects of prior season vaccination; end-of-season analysis of VE for the two most common vaccine types and effects of partial or prior season vaccination is planned. Third, the observational study design has greater potential for confounding and bias than do randomized clinical trials. However, a recent study found that the study design used by the U.S. Flu VE Network produced unbiased VE estimates when applied to analysis of data from randomized placebo-controlled trials (10). In this interim report, adjustment for age, study site, and potential confounding factors identified in previous studies resulted in adjusted estimates that were similar to crude estimates, although final estimates will adjust for additional potential confounders, such as chronic medical conditions, for which information was not available for interim estimates. Finally, end-of-season VE estimates could change as additional patient data become available or if there is a change in circulating viruses late in the season. Also, the VE estimates in this report are limited to the prevention of outpatient medical visits, rather than more severe illness outcomes, such as hospitalization or death; additional studies to measure VE against more severe outcomes are warranted.

Annual vaccination against circulating influenza viruses remains the best strategy for preventing illness from influenza. This report highlights the value of seasonal influenza vaccination and supports ongoing vaccination efforts for all persons aged ≥6 months. Antiviral medications continue to be an important adjunct in the treatment and control of influenza and should be used as recommended, regardless of patient vaccination status.

What is already known on this topic?

The influenza A (H1N1)pdm09 (pH1N1) virus that emerged to cause a pandemic in 2009 has continued to circulate in the United States and has been included as the H1N1 component of all seasonal influenza vaccines since the pandemic. Annual vaccination provides the best protection against circulating influenza viruses and is recommended for all persons aged ≥6 months. Estimates of seasonal influenza vaccine effectiveness (VE) for preventing medically attended illness caused by pH1N1 influenza viruses have ranged from 50% to 80% in previous seasons.

What is added by this report?

Based on data from 2,319 children and adults with acute respiratory illness enrolled in the U.S. Influenza Vaccine Effectiveness Network during December 2, 2013–January 23, 2014, the overall VE (adjusted for study site, age, sex, race/ethnicity, self-rated health, and days from illness onset to enrollment) for all ages against influenza A and B virus infection associated with medically attended acute respiratory illness was 61% (95% confidence interval = 52%–68%). Against the predominant influenza pH1N1 virus, VE for all ages was 62%, with similar protection from medically attended illness across age groups.

What are the implications for public health practice?

The 2013–14 seasonal influenza vaccine provides substantial protection against pH1N1 influenza virus, which has been the predominant influenza virus this season. Persons aged ≥6 months who have not yet received the 2013–14 influenza vaccine should be vaccinated. Evidence of protection offered by the 2013–14 influenza vaccine supports the public health benefit of the recent expansion of annual seasonal influenza vaccination to all persons aged ≥6 months.

## Figures and Tables

**FIGURE:**
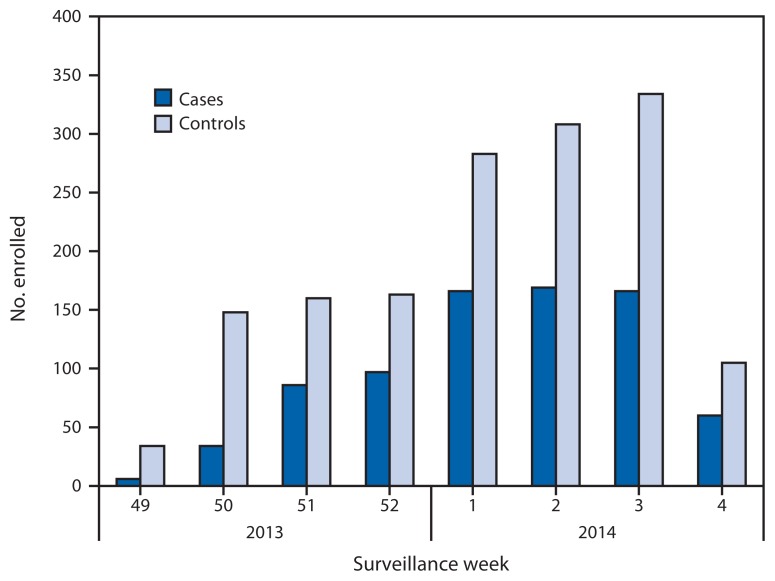
Numbers of influenza-positive, medically attended, acute respiratory illness cases and influenza-negative, acute respiratory illness controls, by week of illness onset — U.S. Influenza Vaccine Effectiveness Network, United States, December 2, 2013–January 23, 2014* * Week 4 only includes patients with completed laboratory tests and thus does not reflect all enrolled patients during that week across study sites.

**TABLE 1:** Selected characteristics for enrolled patients with medically attended, acute respiratory illness, by influenza test result status and seasonal influenza vaccination status — U.S. Influenza Vaccine Effectiveness Network, United States, December 2, 2013–January 23, 2014

	Test result status					Vaccination status			
		
	Influenza-positive		Influenza-negative			Vaccinated*			
					
Characteristic	No.	(%)	No.	(%)	p-value†	No.	Total	(%)	p-value
**Overall**	**784**	**(34)**	**1,535**	**(66)**		**999**	**2,319**	**(43)**	
**Study site**					<0.001				0.03
Michigan	110	(24)	342	(76)		195	452	(43)	
Pennsylvania	196	(36)	354	(64)		209	550	(38)	
Texas	129	(34)	250	(66)		165	379	(44)	
Washington	131	(28)	341	(72)		228	472	(48)	
Wisconsin	218	(47)	248	(53)		202	466	(43)	
**Sex**					0.06				0.16
Male	343	(36)	608	(64)		393	951	(41)	
Female	441	(32)	927	(68)		606	1,368	(44)	
**Age group**					<0.001				<0.001
6 mos–8 yrs	120	(24)	378	(76)		236	498	(47)	
9–17 yrs	52	(26)	150	(74)		61	202	(30)	
18–49 yrs	360	(40)	536	(60)		280	896	(31)	
50–64 yrs	195	(41)	286	(59)		241	481	(50)	
≥65 yrs	57	(24)	185	(76)		181	242	(75)	
**Race/Ethnicity** §					0.001				0.001
White	626	(36)	1,113	(64)		769	1,739	(44)	
Black	61	(29)	153	(71)		64	214	(30)	
Other race	44	(23)	145	(77)		88	189	(47)	
Hispanic	52	(30)	121	(70)		76	173	(44)	
**Self-rated health status** ¶					0.72				0.29
Fair or poor	57	(34)	112	(66)		82	169	(49)	
Good	165	(31)	363	(69)		241	528	(46)	
Very good	315	(35)	590	(65)		378	905	(42)	
Excellent	246	(34)	469	(66)		298	715	(42)	
**Illness onset to enrollment (days)**					<0.001				0.01
<3	360	(45)	447	(55)		316	807	(39)	
3–4	264	(31)	583	(69)		372	847	(44)	
5–7	160	(24)	505	(76)		311	665	(47)	
**Influenza test result**									
Negative	—	—	1,535	—		769	1,535	(50)	
Influenza B–positive	6	—	—	—		4	6	(67)	
Influenza A–positive	778	—	—	—		221	778	(28)	
A (H1N1)pdm09	742	—	—	—		207	742	(28)	
A (H3N2)	13	—	—	—		4	13	(31)	
A subtype pending	23	—	—	—		10	23	(43)	

* Defined as having received ≥1 dose of vaccine ≥14 days before illness onset. According to medical record, to date, 93% of participants had been vaccinated with inactivated influenza vaccines. A total of 56 participants who received the vaccine ≤13 days before illness onset were excluded from the study sample.

† The chi-square statistic was used to assess differences between the numbers of persons with influenza-negative and influenza-positive test results, in the distribution of enrolled patient and illness characteristics, and in differences between groups in the percentage vaccinated.

§ Enrollees were categorized into one of four mutually exclusive racial/ethnic populations: white, black, other race, and Hispanic. Persons identified as Hispanic might be of any race. Persons identified as white, black, or other race are non-Hispanic. The overall prevalences calculated included data from all racial/ethnic groups, not just the four included in this analysis. Race/ethnicity data were missing for four enrollees.

¶ Data on self-rated health status were missing for two enrollees.

**TABLE 2:** Number and percentage receiving 2013–14 seasonal influenza vaccine among 2,319 outpatients with acute respiratory illness and cough, by influenza test result status, age group, and vaccine effectiveness* against all influenza A and B and against influenza A (H1N1)pdm09 — U.S. Influenza Vaccine Effectiveness Network, United States, December 2, 2013–January 23, 2014

							Vaccine effectiveness			
			
	Influenza-positive			Influenza-negative			Unadjusted		Adjusted	
				
Influenza type/Age group	No. vaccinated	Total	(%)	No. vaccinated	Total	(%)	(%)	(95% CI)	(%)	(95% CI)
**Influenza A and B**										
**Overall**	**225**	**784**	**(29)**	**774**	**1,535**	**(50)**	**(60)**	**(52–67)**	**(61)**	**(52–68)**
**Age group**										
6 mos–17 yrs	41	172	(24)	256	528	(48)	(67)	(51–78)	(67)	(51–78)
18–49 yrs	76	360	(21)	204	536	(38)	(56)	(41–68)	(60)	(44–71)
50–64 yrs	73	195	(37)	168	286	(59)	(58)	(39–71)	(60)	(39–73)
≥65 yrs	35	57	(61)	146	185	(79)	(58)	(19–78)	(52)	(2–77)
**Influenza A (H1N1)pdm09**										
**Overall**	**207**	**742**	**(28)**	**774**	**1,535**	**(50)**	**(62)**	**(54–69)**	**(62)**	**(53–69)**
**Age group**										
6 mos–17 yrs	40	168	(24)	256	528	(48)	(67)	(51–78)	(67)	(51–78)
18–49 yrs	70	339	(21)	204	536	(38)	(58)	(42–69)	(61)	(45–72)
50–64 yrs	67	184	(36)	168	286	(59)	(60)	(41–73)	(62)	(42–75)
≥65 yrs	30	51	(59)	146	185	(79)	(62)	(26–80)	(56)	(7–79)

**Abbreviation:** CI = confidence interval.

* Vaccine effectiveness was estimated as 100% × (1 – odds ratio [ratio of odds of being vaccinated among outpatients with influenza-positive test results to the odds of being vaccinated among outpatients with influenza-negative test results]); odds ratios were estimated using logistic regression.

## Footnotes

* The U.S. Flu VE Network sites and the date enrollment began were as follows: Group Health Cooperative (Seattle, Washington) (December 9, 2013); the Marshfield Clinic Research Foundation (Marshfield, Wisconsin) (December 23, 2013); the University of Michigan School of Public Health (the University of Michigan School of Public Health, partnered with the University of Michigan Health System, Ann Arbor, and the Henry Ford Health System, Detroit, Michigan [December 9, 2013]); the University of Pittsburgh Schools of the Health Sciences and UPMC (Pittsburgh, Pennsylvania) (December 2, 2013); and Baylor Scott and White Health, Texas A&M University Health Sciences Center College of Medicine (Temple, Texas) (December 10, 2013).

† An influenza vaccine locator tool is available at http://flushot.healthmap.org/?address.

§ Persons at higher risk include 1) children aged <2 years; 2) adults aged ≥65 years; 3) persons with chronic pulmonary conditions (including asthma); cardiovascular disease (except hypertension alone); renal, hepatic, or hematologic (including sickle cell) disease; metabolic disorders (including diabetes mellitus); or neurologic and neurodevelopment conditions (including disorders of the brain, spinal cord, peripheral nerve, and muscle, such as cerebral palsy, epilepsy [seizure disorders], stroke, intellectual disability [mental retardation], moderate to severe developmental delay, muscular dystrophy, or spinal cord injury); 4) persons with immunosuppression, including that caused by medications or by human immunodeficiency virus infection; 5) women who are pregnant or postpartum (within 2 weeks after delivery); 6) persons aged ≤18 years who are receiving long-term aspirin therapy; 7) American Indians/Alaska Natives; 8) persons who are morbidly obese (i.e., body mass index ≥40); and 9) residents of nursing homes and other chronic-care facilities.

¶ Influenza vaccination coverage estimates for the 2013–14 season are available at http://www.cdc.gov/flu/fluvaxview/index.htm.

** A complete summary of guidance for antiviral use is available at http://www.cdc.gov/flu/professionals/antivirals/summary-clinicians.htm.
